# Evaluating the effect of sepsis and septic shock on myocardial functions by echocardiography and serum biomarker level in peripheral veins and coronary sinus

**DOI:** 10.1186/cc14216

**Published:** 2015-03-16

**Authors:** M Soliman, A Alazab, R El Hossainy, M Nirmalan, H Nagy

**Affiliations:** 1 Cairo University, Cairo, Egypt; 2 University of Manchester, UK

## Introduction

Sepsis is a leading cause of death in critically ill patients despite the use of modern antibiotics and resuscitation therapies. Biomarkers and cardiovascular changes have an important place in this process. Myocardial depression occurs in 40% of septic patients.

## Methods

Twenty patients (group I) with sepsis or septic shock were included and 10 patients (group II) served as the nonseptic group. Group I morbidity and mortality at day 28 in the ICU were targeted as the endpoint. Laboratory investigations, APACHE IV, SAPS II and SOFA scores were calculated. Biomarkers IL-1α, IL-1β, IL-6, IL-10, TNFα, CRP, NT-proBNP and troponin level were estimated on admission and day 7 in the peripheral vein (PV) and coronary sinus (CS). Transthoracic echocardiography and tissue Doppler imaging was done on admission and on day 7.

## Results

Comparing group I versus group II, the mortality rate was 45%, and there was a statistically significant difference for temperature (*P *= 0.001), HR (*P *= 0.001) and WBC count (*P *= 0.01) on admission. Upon comparing survivors versus nonsurvivors in group I there was a statistical difference in HR on day 7 (*P *= 0.02), successful vasopressor withdrawal (*P *= 0.02), P/F ratio (*P *= 0.02) and ScVO_2_ on day 7 (*P *= 0.03). Regarding IL-1α, IL-1β, TNFα and troponin I there was no statistical significant difference between groups I and II but IL-6, IL-10 and CRP showed statistically significant difference on admission PV and CS. Pro-BNP shows statistically significant difference in all CS samples between septic and nonseptic groups. Regarding echo upon comparing the survivors versus nonsurvivors, E'd/t on day 0 shows a statistically significant difference between both groups. SAPS II and seventh-day SOFA are good predictive scores for mortality in sepsis.

## Conclusion

Diastolic dysfunction was seen in 90% of patients. Fever, HR, and WBC counts are still good early indicators for diagnosis of sepsis. Vasopressor withdrawal on the seventh day was a good predictor for survival. Admission serum IL-6, IL-10 and CRP from PV were better indicators for sepsis than IL-1, pro-BNP and troponin I. Admission TNFα and seventh-day IL-6 levels were highly prognostic for mortality. CS samples proved that NT pro-BNP is a good indicator for sepsis diagnosis and a good predictor for survival. TNFα from CS samples was also a good predictor of mortality. SAPS II and a slower E'd/t on admission was a good predictor of mortality.
